# An Experience of Public Dental Care during the COVID-19 Pandemic: Reflection and Analysis

**DOI:** 10.3390/ijerph18041915

**Published:** 2021-02-16

**Authors:** Orsolya Nemeth, Mercedesz Orsos, Fanni Simon, Peter Gaal

**Affiliations:** 1 Department of Community Dentistry, Faculty of Dentistry, Semmelweis University, 1088 Budapest, Hungary; orsos.mercedesz@dent.semmelweis-univ.hu (M.O.); simon.fanni@dent.semmelweis-univ.hu (F.S.); 2 Health Services Management Training Centre, Semmelweis University, 1125 Budapest, Hungary; gaal@emk.sote.hu

**Keywords:** infection control, COVID-19, pandemic, community dentistry, dental public health, dental management

## Abstract

Since its emergence in China, the COVID-19 pandemic has become the number 1 health challenge in the world with all affected countries trying to learn from each other’s experiences. When it comes to health services, dental care does not seem to be a priority area, despite the fact that it is among the highest risk medical specialisations in terms of spreading the infection. Using the Department of Community Dentistry of Semmelweis University as a case study, the objective of this paper is to introduce and analyze the system and organizational level measures, which have been implemented in dental care in Hungary during the first months of the COVID-19 outbreak. The system level measures to promote social distancing, to reduce the use of health services and to protect high risk health professionals, together with the deployment of protective equipment and the reorganization of patient pathways at the organizational level proved to be effective in keeping the outbreak in control. There are two, less frequently mentioned ingredients of successful coping with the COVID-19 challenge. First, mental health support is at least as important as physical protection. Second, most of the interventions do not require big financial investments, but behavioural change, which in turn requires leadership and change management skills.

## 1. Introduction

The Corona Virus Disease 2019 (COVID-19, SARS-CoV-2) pandemic took the leaders of many countries all over the world by surprise, despite other similar epidemics of the past, which have served as a warning to prepare for the worst for only a handful of countries that had been actually hit by Severe Acute Respiratory Syndrome (SARS) and Middle East Respiratory Syndrome (MERS), and eventually successfully contained the outbreaks [1,2]. After the initial period of confusion, blundering and even denial, governments have implemented unprecedented social distancing measures in a desperate attempt to slow down the spreading of the infection and “flatten the curve” [3]. The case of Italy has shown to everyone how the uncontrolled transmission of the novel coronavirus is able to quickly overwhelm health services and produce untimely deaths in great numbers due to the lack of sufficient critical care capacities [4,5]. Learning from the Italian example, the more fortunate countries of Europe have understood that apart from the implementation of strict social distancing measures, the key area of combating the epidemic is the reorganization of health service delivery to decrease avoidable mortality until we have a treatment of proven effectiveness, or an effective vaccine [6,7,8].

In Hungary, the first two patients with the novel SARS-CoV-2 infection were officially diagnosed and reported on 4 March 2020. On March 11 the Hungarian Government declared a state of emergency [9], which was followed by a number of social distancing measures, including school closures, restrictions on occasions of gatherings, retail trade limitations and eventually a curfew with few exceptions [10]. As far as the health sector is concerned, health workers over the age of 65 or with chronic diseases have been withdrawn from direct patient care, non-emergency care has been suspended, elective surgeries have been postponed and the Ministry of Human Capacities have ordered hospitals to empty as many as 50% of their bed capacity by 19 April, as a preparation for a surge in the number of infected patients requiring inpatient care [11].

In this paper, we examine the rationale behind the reorganization of health service delivery, as a response to the COVID-19 pandemic, introduce and analyze the already implemented measures in Hungary, focusing on dental care. We illustrate our analysis with the experiences of the Department of Community Dentistry of Semmelweis University, Budapest, as a case study. Finally, we conclude with some recommendations and lessons learnt.

### 1.1. Epidemic Concerns in Dental Care

Dentistry is among the highest risk specialties in terms of contracting and spreading the virus. Dentists, dental nurses and assistants are exposed to the SARS-CoV-2 infection on account of aerosol-generating medical procedures (AGMPs) [12]. The physical proximity of dental health workers to patients (approximately 25–30 cm) and the length of the various treatments is considerable; therefore, exposure to the disease is extremely high.

According to Judson et al., AGMPs can be grouped into two categories: procedures that mechanically create and disperse aerosols, and procedures that induce the patient to produce aerosols [13]. Almost all dental procedures involving the use of dental handpieces, ultrasonic scalers, air polishing devices and air abrasion units mechanically produce aerosols. The propelling force of a high-speed dental drill and the cavitation effect of an ultrasonic scaler, combined with a water spray can generate numerous airborne particles derived from blood, saliva, tooth debris, dental plaque, calculus, and restorative materials.

### 1.2. System Level Responses to the Pandemic: The Reorganization of Dental Care

To defuse the health system “transmission bomb”, it is inevitable to reorganize care to protect health workers and patients from the infection, while COVID-19 patients need to be properly cared for at the same time. One obvious option to do that is to reduce the number of patient-doctor encounters by temporarily suspending services, which can be postponed without harming the patients. Several countries have considered such restrictions among the first measures to slow down virus transmission [14,15]. Although specific measures vary from country to country, most guidelines agree in that non-urgent, elective treatments should be postponed, including cosmetic treatments, elective surgeries and orthodontic treatments [16]. However, while in some countries, such as the UK, Denmark, Norway, Portugal, Poland and Israel the suspension was mandatory, in others, such as Belgium, France, the Netherlands and Bulgaria it was a recommendation, and in Germany, Austria, Sweden, Slovakia and Malta legislative bodies only gave suggestions for increased occupational safety and infection control [15,16,17,18].

In Hungary, these treatment limitations were compulsory, mandated by central regulations. A national emergency was declared by the government on 11 March, just 7 days after the confirmation of the first two official COVID-19 cases. On 14 March, a ministerial order restricted the operation of primary dental care to emergency procedures only, all other interventions were prohibited [19]. At that time, only 25 people were registered with coronavirus infection in the country. Subsequently, on 17 March, specialist dental care was also strictly reduced to emergency procedures only [20]. Private sector providers have also complied with the legal restrictions and either reorganized their activities to provide only emergency services or simply suspended their operations altogether. This situation has placed a very heavy burden on the shoulders of public providers, which have already been struggling with funding difficulties and crowding.

While it is relatively easy to say that non-emergency care has to be suspended, the operationalization of what is considered deferrable is not that straightforward. Diseases and conditions, which threaten the life of patients, or result in permanent health damage without immediate action, are not in question. However, what about others, which are not life or health threatening now, but will be some time in the future, as the disease progresses?

Fortunately, the category of urgent dental services has already been precisely defined and regulated in Hungary, and a positive list has been promulgated in a ministerial decree [21]. It is interesting to note that this regulation dates back to the 1994–1998 governmental period, when the coverage of dental services in the social health insurance system was curtailed, e.g., tooth preserving dental services were excluded [22,23]. One exception was urgent care, and the health government had to operationalize what is considered urgent, because it was subject to intensive debates between dental providers and the social health insurance fund.

Medical Interventions, which Belong to the Category of Urgent Dental Care (Article 4, Section (2) and Annex 2 of Decree No. 48/1997. (XII.17.) NM of Minister of Welfare)

- Primary care for injured teeth (replacement, splinting, tooth extraction).
- Treatment of dental inflammations (trepanation, tooth extraction).
- Conservative treatment of alveolitis, pericoronitis.
- Abscess opening in oral cavity.
- Removal of a foreign body that obstructs swallowing and/or breathing.
- Medication for acute inflammatory diseases of the oral mucosa and lips.
- Reducing the dislocated jaw.
- Resting the broken jawbone.
- Attenuation trigeminal neuralgia by local anesthesia.
- Relief of bleeding in or around the mouth of any origin (tampons, bandages, application anticoagulants, sutures).

While the postponement of non-emergency care is a very important measure to bring the COVID-19 epidemic under control, they do not eliminate all the risks of the virus transmission, since patients needing urgent dental interventions still need to be attended. Furthermore, the turnover of such patients are expected to be high, since they disproportionately fall on the publicly funded dental service providers due to the closure of private practices, and inevitably increases over time with some of the unattended cases deteriorating to the point of becoming an emergency. In addition, the remaining operational care capacities is decimated with the withdrawal of high risk health workers from direct patient care, which further aggravates the human resources shortages the health system has already been experiencing for the past 15 years [24]. Among these strained conditions it is crucial to organize service delivery in a way, which best protects patients and dental health workers alike. On 27 March, the Council of European Dentists (CED) issued a press release calling on the European Commission and governments of Member States to support professionals working in the public and private dental sector, both financially and in terms of providing protective equipment [25].

In Hungary, ensuring the highest possible safety by the timely identification of infected patients, by the appropriate use of protective equipment and by the organization of patient pathways and the coordination of care has been the responsibility of the management of individual service providers [26,27].

## 2. Materials and Methods

### 2.1. Responses at the Organizational Level: The Case of the Department of Community Dentistry

The Department of Community Dentistry, Semmelweis University, Budapest (Hungarian acronym FSZOI) is the largest and most popular, publicly funded dental clinic in Hungary. It provides the full range of dental treatments, including 24-7 emergency care, dental surgery, periodontics, implants, orthodontics, pedodontics, endodontics, prosthodontic, cosmetic dentistry, services for patients with special needs and dental hygiene. The departmental building houses 32 dental chairs on which patients are treated each workday (Monday to Friday) from 8 am to 8 pm. Emergency dental care is available in the evenings from 8 pm to 8 am and also all day on the weekends and holidays. The institution takes part in both undergraduate and postgraduate training, and has a practice laboratory for dental technicians. Its departments are structured according to the requirements of the principles of community dentistry, and have collaborations with a wide range of institutions to care for special patient groups, as well, such as children and patients with special needs.

The 79 dentists and 61 dental assistants/hygienists provide dental care for 165,000 patients, and train about 450 students every year. Apart from complying with the COVID-19 regulations, all the introduced measures focused on the safety of patients and the employees. As of 17 April, the coronavirus crisis in Italy has already killed 119 doctors and another 87 health workers among the close to 17,000 who got infected. There have been 12 dentists among the dead [28].

In line with the order of the Minister for Human Capacities, all planned, elective treatments were postponed from 14 March. Like many other countries, Hungary temporarily closed down all educational institutions, therefore we also postponed the screening of children and treatment of patient living with disabilities. Online university education started on 23 March, which lifted the workload related to the practice training of dental students.

### 2.2. The Deployment and Use of Protective Equipment and Procedures

Without the health and safety of the health professionals, dental teams would be unable to guarantee continued patient care. The strict implementation of disinfection procedures and the provision and proper use of the necessary protective equipment, along with the proper training of staff, are crucial to decrease the risk of infection [29]. Every health worker is recommended to use soap, alcohol-based rubs, and wear a facemask, gown, gloves and goggles [30].

The National Health Commission of the People’s Republic of China introduced a handwashing procedure called “two before and three after”. It recommends handwashing with running water and detergents in proper times: before touching patients, before every aseptic and invasive treatment, and after touching the patients and the patients’ surroundings and items. According to a recent COVID-19 study, 66.1% of the followed health workers washed their hands 10 times a day and 22.1% of them applied protective skin care after handwashing. While hand and body disinfection is crucial to avoid contamination, they have side-effects, such as skin and mucosa irritation, and contact dermatitis. Furtherly, these destructions can become a source of seconder infections [31].

As far as disinfection agents are concerned, SARS-Cov-2 has been shown to be inactivated by ultraviolet light, hot water bath for 30 min, chlorine-containing disinfectants, 60–70% ethanol, 0.5% hydrogen peroxide, 0.1% and sodium hypochlorite, while 0.2% benzalkonium chloride and 0.02% chlorhexidine digluconate has proven to be less effective [31,32,33]. In our department alcohol-based liquid disinfectants and wipes are used for surface disinfection, sodium perborate-, and tetraacetylethylenediamine-bearing formulations for the disinfection of tools, while the hand sanitizers are alcohol-based rubs.

Health workers should wear duckbill or cup-shaped masks and respirators, which do not collapse against the mouth. The most commonly engaged respirator type is N95. This is an American standard managed by the National Institute for Occupational Safety and Health of the Centers for Disease Control and Prevention. Europe recommends a filtering face piece score (FFP), which comes from a European standard (EN) maintained by CEN, the European Committee for Standardization. According to the EN, FFP2 and FFP3 are the gold standards. Gowns are usually for single use and disposable. Protective goggles can be reusable or disposable. Both of them require a good seal to facial skin and have clear plastic lenses. Face shields are made of clear plastic to provide good transparency. They are mainly re-usable, but disposable face shields are also an option. [34,35].

In the dentistry department, safety regulations and procedures are based on these principles of advanced hygiene protocol. The primary recommendations of hand hygiene are strictly mandatory for each staff member. The protective gear of the staff consists of a face-mask, a gown, gloves, shields and goggles. Table 1. shows the required PPE for the various personnel and for the patients at different locations along the patient pathway.

### 2.3. Care Pathways: Identification and Separation of the Infected

**Figure 1:**
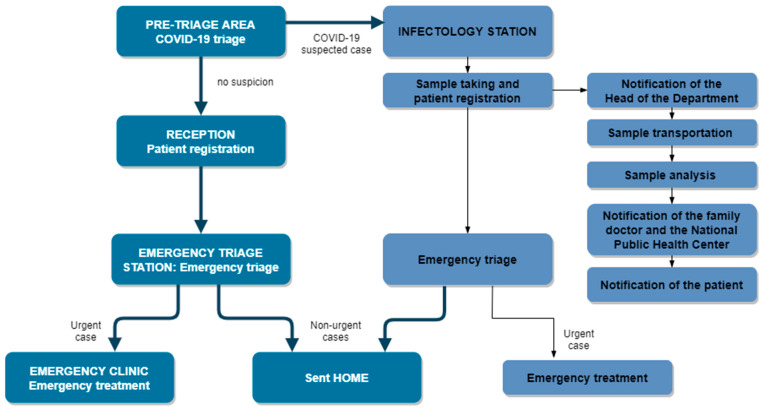
Patient pathways in the Department of Community Dentistry of Semmelweis University.

On March 23, Semmelweis University introduced a triage procedure to identify the potentially infected. Every patient (Figure 1) has to go through a COVID-19 triage before registering in the clinical report system: temperature measurement, health status, history and recent travels in the past 14 days are assessed. If, based on the questionnaire and the symptoms, the patient is identified as a suspected case, the COVID-19 test is indicated. The university uses both quick tests and polymerase chain reaction (PCR) laboratory tests. Those patients who show symptoms or carry suspicion of being infected get tested by both an immune serological blood-tests and PCR tests. The National Public Health Center and the patient’s family doctor are notified about the suspected case.

The patient pathways are completely separated in the case of COVID-19 suspicion. The infectology station is located on a separate floor of the institute, where only patients suspected of being infected are admitted, using an isolated elevator, and there is a separate elevator for the staff members. The sampling for the laboratory tests, the emergency triage and the necessary treatments are all performed in the infectology station for suspected cases. In this setting the patient also wears protective gear and the dental team has special masks with positive pressure and PPE.

All test results appear in the electronic hospital information system within a few hours (maximum 24 h), and the results are also uploaded to the eHealth cloud (the so-called National eHealth Infrastructure), which is also accessible to the patient and the patient’s family doctor.

Patients with no suspicion of infection are registered at the reception and proceed to an emergency triage station to decide whether their problem claims urgent service, or not. If the case does not belong to any of the urgent care categories, shown before—for example, if the patient desires tooth bleaching, or new ceramic veneers—they are politely asked to go home, and return when the restrictions on service deliveries are lifted. Prescriptions are provided by the triaging dentist, if needed. If the treatment cannot be postponed without threatening the life or health of the patient, then the patient goes to the emergency clinic, where the necessary interventions are performed. To decrease the risk of infection from unidentified, asymptomatic COVID-19 cases, there is a recommendation in effect to minimize aerosol generating procedures (3-way syringe, turbine, ultrasonic scaler) during treatment in the pandemic period [32].

In line with the expansion of the COVID-19 epidemic, the emergency services were reorganized in the department on 6 April. In order to rationalize the use of disposable protective equipment, three shifts were reduced to two shifts without eliminating opening hours to ensure the appropriate protective equipment (FFP2, shields, etc.). The current teams consist of two oral surgeons, one specialist of operative dentistry, one specialist of pedodontics/orthodontics and one experienced dentist in triage equipped with full protective gear, all of whom are accompanied by five dental assistants. There are 8 such teams in rotation, each working two days in a row for 12 h per day, followed by a period of rest until their next turn. This rest period roughly equals to the incubation time of the virus, which makes it more likely that the potentially infected colleagues can be filtered out. For the night shifts, there are 3 teams with one dentist, one dental resident and 2 dental assistants per team, which are also in rotation.

**Figure 2:**
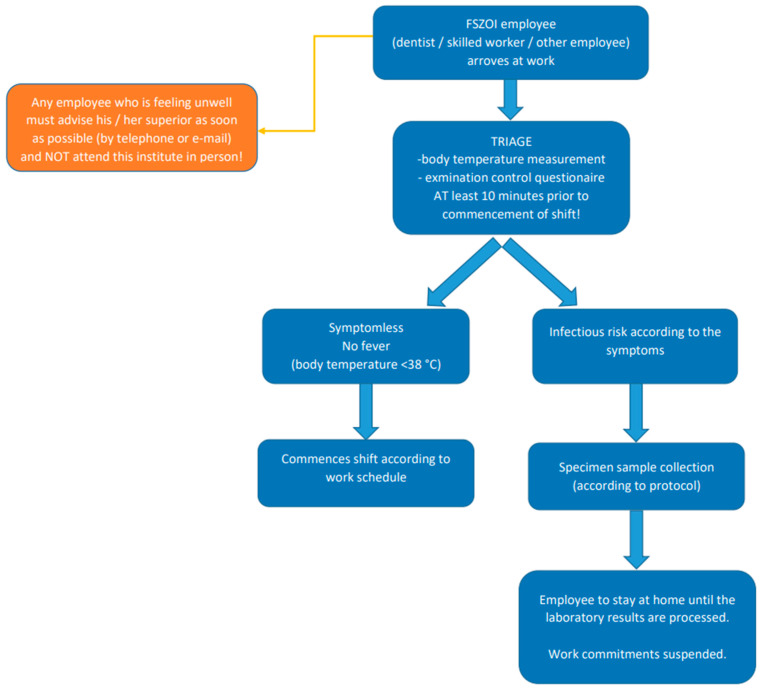
Employee screening in the Department of Community Dentistry of Semmelweis University (FSZOI).

All employees undergo a COVID-19 screening procedure (Figure 2) and are tested regularly every two weeks. Both immune serological blood-tests and PCR tests are compulsory regardless of the symptoms. If any member of the 8 plus 3 teams is found to be positive for COVID-19 during the regular testing, the whole team is temporarily taken out of the system.

Among the team members the oral surgeons are assigned to treat the suspected COVID-19 cases. Once a suspected case is attended, the oral surgeon and the dental assistant are not allowed to return treating other patients not suspected to be infected.

## 3. Results

### Capacities and Patient Turnover

**Figure 3:**
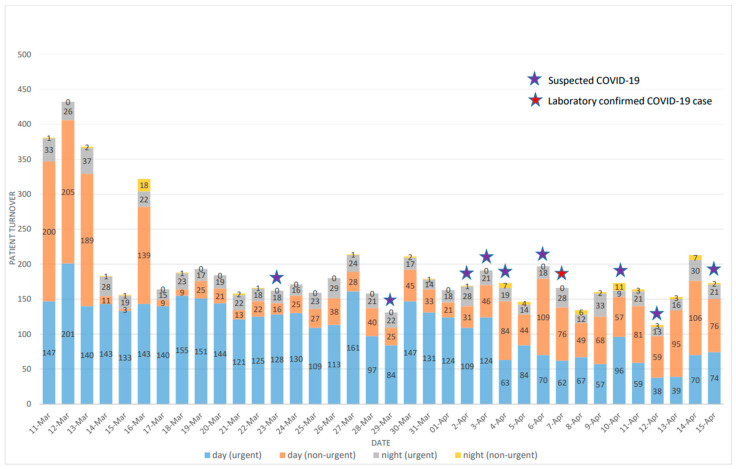
Patient turnover in the Department of Community Dentistry of Semmelweis University, 11 March to 15 April 2020.

The average number of patients admitted to our Department was around 300–350 persons per day. This number includes emergency cases from both the day and night shifts, as well as patients receiving specialist dental care in the department. The first legal restriction on primary dental care on 14 March had not affected the patient turnover yet. The 17 March restriction of specialist dental care caused a visible relapse in the number of daily admissions. Figure 3 shows that the average number of patients visiting the Department suddenly dropped after 16 March from over 300 to less than 200 a day and reached its lowest on 12 April with 115 a day. From 17 March, there were only two days when the daily number of patients exceeded 200—the 27 and 30 March—which might be related to the partial curfew announced on 27 March and effective of 28 March, although visiting a doctor has always been an exemption. The much lower numbers in between are attributable to the weekend (Saturday and Sunday), which seem to be a general pattern for the study period. Still, with these numbers, the Department of Community Dentistry of Semmelweis University remained one of the most frequented dental institution in the country, and the patients presented themselves from a much broader geographical area (90.2% of the patients, 316 per day) than the usual catchment area of the Department (9.8% of the patients, 34 per day).

It is a crucial question of emergency dental care, whether the showing of a patient is medically justified. In the department the triage-system filtered out 1592 (29.3%) non-urgent cases out of the total 5435 patients between 16 March and 15 April 2020.

Figure 3 also shows the trend of the number of suspected COVID-19 cases and the number of PCR confirmed cases after 16 March. As we can see during this period there were only 11 suspected cases, out of which only 2 proved to be SARS-Cov-2 positive by the PCR laboratory test. This finding is not at all surprising in light of the dynamics of the COVID-19 outbreak in Hungary.

**Figure 4:**
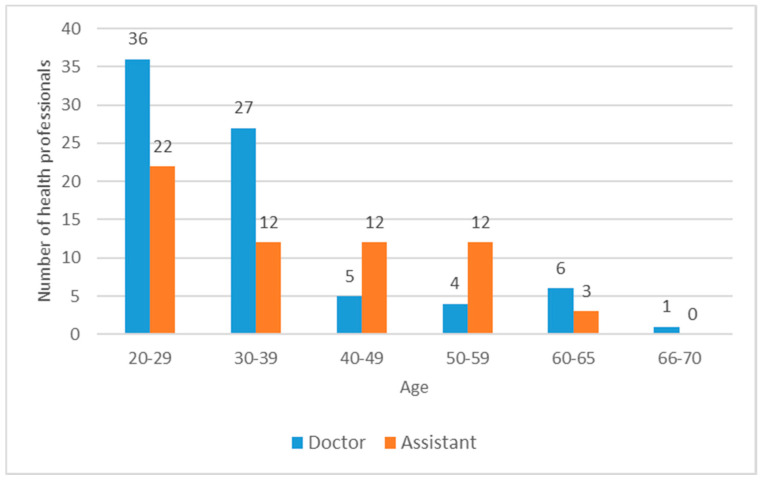
Age distribution of health professionals of the Department of Community Dentistry of Semmelweis University as of 11 March 2020.

Even with a relatively low patient turnover, capacities can be quickly overwhelmed if staff members are temporarily or permanently lost due to the disease. Of course, some of these losses are inevitable to prevent further short or long term losses. According to the order of the Minister of Human Capacities, health professionals over the age of 65 and with certain chronic diseases had to be withdrawn from contact patient care. As shown in Figure 4, there was only 1 dentist above the age of 65, whom we had to withdraw from direct patient care and there was another one with a chronic disease. Another 9 staff members (3 doctors and 6 dental assistants) took an unpaid leave to care for their elderly relatives and/or children, living in the same household. The increasing fear and anxiety among patients and staff members could also detrimentally affect the willingness to work, and could threaten the integrity of the teams through the generation of panic among the colleagues. In the Department of Community Dentistry 1 dentist and 1 dental assistant were asked to go on unpaid leave after the news of the virus outbreak reached public awareness. Although Semmelweis University had immediately set up mental health support groups to help resolve employees’ fears and anxieties, some colleagues were not able to handle the situation in fear of theirs or their families safety.

As far as the SARS CoV-2 infection of staff members are concerned, two dentists produced a positive serology test, but later proved to be PCR negative, and the partner of one other colleague was found infected, which was fortunately not transferred to her. All these means that for the most active phase of the outbreak, we lost only 6 doctors (7.6%) and 7 assistants (11.5%) out of the 79 and 61, respectively, which allowed us to comfortably manage the reduced patient load.

We have started collecting feedback from employees. The first satisfaction questionnaire was filled in by 43.6% (61) of our health professionals. 73.7% (45) of the respondents were absolutely satisfied with the safety of working conditions. Since then, an increasing number of similar questionnaires on the subject have been completed.

## 4. Discussion and Recommendations

Although dentistry does not count a frontline medical specialization of this current epidemic, it plays an important part to maintain essential services by handling dental emergency cases, but also represents a valuable capacity that can be reallocated if need arises. Just as any other health services, it is a potential high risk source of the epidemic, as well, which justifies priority to be given to the protection of dental health workers and patients alike.

The case of Hungary and the Department of Community Dentistry of Semmelweis University has shown that with appropriate and timely measures, both the minimum necessary dental services can be maintained during the expansive stage of the epidemic, and the provision of dental care can be avoided to become a source of spreading the disease. Together with the deployment and use of protective equipment, such as disposable masks, shields, clothing, gloves and hat (some of which are also applicable to patients, not just health workers), as well as the rigorous implementation of simple disinfection procedures, such as handwashing (not just at the workplace, but at home), clearly set and enforced patient pathways and processes are crucial to weather the COVID-19 pandemic with minimal loss of lives and health. These latter measures are often simple, such as keeping the appointment times, minimizing patient interactions in the waiting room, minimizing aerosol production by the limited use of water-cooled handpieces (i.e., only when absolutely necessary), or the follow-up of suspected cases, and do not require big financial investments, but behavioral change, which, to be successful, requires leadership and conscious and continuous change management support [36].

Maintaining the necessary operation during the expansive phase of the outbreak depends on the availability of human resources, whose retaining has been much more threatened by their ability to coping with the situation mentally, than by the infection itself. Mental health support services, therefore, are crucial to be accessible for staff members along with the physical protection against contracting the disease.

## 5. Conclusions

Finally, it is important to note that environmental changes, such as the COVID-19 pandemic, are not only threats to our comfortable and familiar routines, but an opportunity to change for the better. The development of eHealth and teledentistry, whose adoption was slow in Hungary before the outbreak, is one such opportunity. These technologies are an exceptional option to invite older colleagues back to patient care without any risk of contracting the disease. While this would certainly require some effort from all involved parties, such an investment should not be considered a one time COVID-19 special, but a long term commitment to modernize dental care, making it more efficient and accessible for generations of patients to come.

## Figures and Tables

**Table 1:** Internal regulations on protective equipment in the Department of Community Dentistry of Semmelweis University.

Premises	Individuals	Face Mask	Gown	Gloves	Shield	Goggles	Hat
Reception	Receptionist	FFP2	-	+	-	-	-
Pre-triage area (COVID-19 triage)	Dentist triaging	FFP2	Medical PPE protective coverall suit	+	+	+	+
Waiting room adjacent to the infectology station	Patients with suspected infection (COVID-19 patients)	Surgical mask	Medical isolation gown	+	-	-	+
Infectology station	Dentist triaging & treating COVID-19 patients	Positive pressure FFP2	Medical PPE protective coverall suit	+	+	+	+
	Dental assistant treating COVID-19 patients	FFP2	Medical PPE protective coverall suit	+	+	+	+
Emergency triage station	Dentist triaging other (not infected) patients	FFP2	Medical isolation gown	+	+	+	+
Emergency clinic/Oral surgery	Dentist treating other (not infected) patients	FFP2	Medical isolation gown	+	+	+	+
	Dental assistant treating other (not infected) patients	FFP2	Medical isolation gown	+	+	+	+
Waiting room adjacent to the emergency clinic	Non-infected patients	Surgical mask	-	-	-	-	-

## Data Availability

The data sets used and/or analyzed during the current study are available from the corresponding author on reasonable request.
